# A Methodical Needle-Over-Needle Guidance (NNG) Approach for Spinal Anesthesia Using a “Super Introducer”

**DOI:** 10.7759/cureus.81176

**Published:** 2025-03-25

**Authors:** Mahesh Desilva, Jonathan Shen, Cherry Liu, Nick Wixon, Shruthi Krishnamurthy

**Affiliations:** 1 Anesthesiology, Riverside Community Hospital, Riverside, USA

**Keywords:** morbid obese spinal anesthesia, needle-over-needle guidance, post dural puncture headache, spinal anesthesia (sa), super introducer

## Abstract

Post-dural puncture headache (PDPH) often occurs after lumbar puncture during spinal anesthesia, and younger patients tend to be more susceptible to this complication. In this case report, we present a needle-over-needle guidance (NNG) technique utilizing a larger gauge (20G) 3.5” regular spinal needle, referred to as the “Super Introducer,” to guide a longer, smaller gauge (25G) 4.7” spinal needle toward the dura in the midline. The “Super Introducer,” which is longer than the standard introducer needles found in spinal kits, allows for deeper engagement with the interspinous ligament and provides directional guidance to successfully advance the longer spinal needle into the dural sac. Additionally, we demonstrate an innovative approach for methodically advancing the “Super Introducer” safely, based on a careful examination of the relationship between the regular and long spinal needles used. This technique resembles the combined spinal-epidural technique but may offer advantages such as being faster, more cost-effective, and less uncomfortable for the patient. Potential variations of this technique include using a long 27G-29G needle over a regular 22G spinal needle or a long 25G needle over a regular 20G spinal needle. NNG techniques like the “Super Introducer” method can aid in achieving successful spinal anesthesia or lumbar puncture with smaller gauge pencil-point needles, particularly in younger, morbidly obese patients. By employing proper technique, this approach can help reduce the risk of PDPH in this population while also minimizing needle bending and the risk of needle breakage.

## Introduction

Soto and Schultetus first introduced the concept of using a regular spinal needle as an introducer for a longer spinal needle [1]. This case report revisits the needle-over-needle guidance (NNG) approach using a “Super Introducer.” We propose a step-by-step technique to safely advance a long 4.7” (120 mm) spinal needle over a regular 3.5” (90 mm) spinal needle “Super Introducer” to reach the dural sac effectively.

Post-dural puncture headache (PDPH) is a common complication of lumbar puncture during spinal anesthesia, with younger patients being particularly vulnerable [2]. According to an obstetric anesthesiology closed claim study published in the 1999 American Society of Anesthesiology newsletter, PDPH accounted for 15% of obstetric claims, making it the third most common claim. Substantial evidence indicates that using atraumatic blunt pencil-point needles significantly reduces the incidence of PDPH compared to cutting needles [3-7]. Additionally, smaller diameter spinal needles are associated with a lower frequency of PDPH [8,9], leading to the common recommendation of using 25G or smaller pencil-point needles for patients at risk of PDPH [10].

However, narrower-gauge spinal needles, particularly the longer 4.7-5” variety, are more challenging to advance due to their tendency to deflect and bend. This issue is further exacerbated in obese patients with increased depth to the intrathecal space, increasing the risk of placement failure [11,12]. Another concern with using these higher gauge needles is the potential for needle breakage [13]. Due to their metallic weakness, long, narrow-gauge needles are more prone to bending and fracturing [14]. Reports of deformation and fracture of narrow-gauge spinal needles have been documented, with three cases associated with obstetric anesthetic practice [15]. A systematic review of spinal needles broken during neuraxial anesthesia identified obese patients with difficult anatomical landmarks and the use of smaller gauge needles (25G or smaller) as having a higher risk of needle fracture [16].

To minimize the risk of needle breakage, employing NNG techniques with non-cutting needles is advisable. The most common NNG approach involves using an introducer needle [17]. Standard introducer needles included in spinal kits are typically 18G-20G and 3.2-3.8 cm long. The larger gauge introducer facilitates the insertion of non-cutting needles and is particularly beneficial when using fine needles of 25G or smaller [18]. However, standard introducer needles are often too short to adequately engage the interspinous ligament in morbidly obese patients, leaving the long spinal needle to traverse a considerable distance to the dura, increasing the risk of deviation and fracture.

Another commonly used NNG technique in clinical practice involves guiding the spinal needle to the dura using an epidural needle, as seen in the combined spinal-epidural (CSE) technique [19].

In this case report, we describe a third NNG technique involving a “Super Introducer” - a larger gauge (20G) 3.5” regular spinal needle used to guide a longer, smaller gauge (25G) 4.7” spinal needle toward the dura in the midline. The “Super Introducer,” which is longer than the standard introducer needles in spinal kits, allows for deeper engagement with the interspinous ligament and provides directional guidance to successfully advance the longer spinal needle into the dural sac.

We also demonstrate a novel method for safely advancing the “Super Introducer” by closely examining the interaction between the regular and long spinal needles. While this technique is not superior to the CSE technique, it may offer advantages such as being faster, more cost-effective, and more comfortable for the patient. Potential variations of this technique include using a long 27G-29G needle over a regular 22G spinal needle [20] or a long 25G needle over a regular 20G spinal needle.

NNG techniques like the “Super Introducer” method can enhance the success of spinal anesthesia or lumbar puncture using smaller gauge pencil-point needles, particularly in younger morbidly obese patients. By employing proper technique, this approach may help reduce the risk of PDPH while minimizing needle bending and the potential for needle breakage.

## Case presentation

A 51-year-old, 5’11”, 300 lb (BMI 42) morbidly obese male patient with a history of moderate persistent asthma was scheduled for left ankle open reduction internal fixation surgery. The patient was identified as a potential difficult airway case due to a short neck, large body habitus, Mallampati 4 classification, and a large beard. With the patient’s consent, the plan was to proceed with spinal anesthesia as the preferred primary mode of anesthesia, supplemented by monitored anesthesia care (MAC) sedation for comfort. General endotracheal anesthesia was designated as the backup plan, and a popliteal sciatic/saphenous nerve block was planned for postoperative analgesia.

The patient was placed in a sitting position in the operating room, and monitors were applied. The L4-5 interspace was approximated using the top of the iliac crest as a landmark. The midline was identified and marked using a curvilinear ultrasound probe. The L3-4 interspace was selected for initial needle entry, and after topical infiltration with 1% lidocaine, a 20G × 3.5” BD Quincke spinal needle “Super Introducer” was inserted approximately 5 cm deep to engage the interspinous ligament. A 25G × 4.7” BD Whitacre spinal needle was then inserted through the Quincke introducer.

When CSF flow was not observed in the Whitacre spinal needle, it was removed, and the 3.5” Quincke “Super Introducer” was cautiously advanced an additional 0.8 cm. These steps were repeated several times until CSF flow was detected in the Whitacre spinal needle, indicating successful entry into the dural sac. A total of 1.4 cc of 0.75% hyperbaric bupivacaine was injected, with confirmation of birefringent CSF flow in the syringe at both the midpoint and end of the spinal injection (Video 1, Video 2).

**Video 1:** Clinical application of the NNG technique using a “Super Introducer” “Super Introducer” = A 3.5" (8.9 cm) 20G Quincke spinal needle that is longer than the standard 1-3/8" (3.5 cm) 20G introducer typically included in spinal kits. NNG, needle-over-needle guidance

**Video 2:** Animated demonstration of the NNG technique using a “Super Introducer” for spinal anesthesia NNG, needle-over-needle guidance

The patient had an uneventful course under spinal anesthesia and MAC sedation. At the conclusion of the surgery, an ultrasound-guided popliteal sciatic and saphenous nerve block was performed to aid in managing postoperative pain.

## Discussion

**Figure 1:**
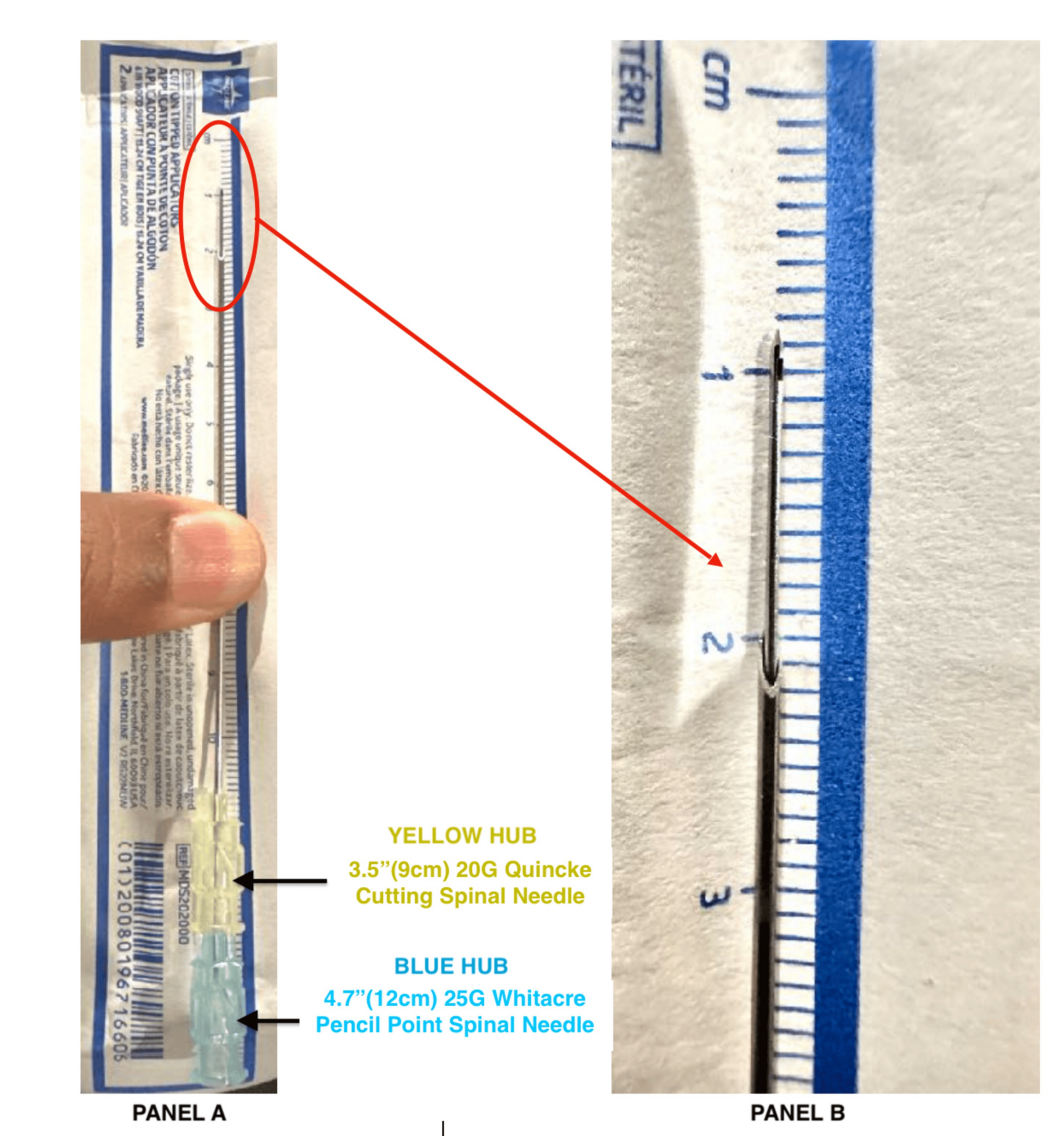
NNG “Super Introducer” technique using a 4.7” 25G BD Whitacre spinal needle (blue hub) fully inserted through a 3.5” 20G BD Quincke spinal needle (yellow hub) (A) A 4.7” (12 cm) 25G BD Whitacre pencil-point spinal needle (blue hub) fully inserted through a 3.5” (9 cm) 20G BD Quincke cutting spinal needle (yellow hub). (B) Magnified view of the distal needle relationship, showing the 4.7” 25G BD Whitacre spinal needle (pencil-point) extending approximately 1.1 cm beyond the 3.5” 20G BD Quincke spinal needle (cutting) when fully inserted. NNG, needle-over-needle guidance

A unique aspect of this NNG case report is the safe, systematic advancement of the long 4.7” BD Whitacre needle over the regular 3.5” BD Quincke needle, referred to as the “Super Introducer.” When fully inserted through the 3.5” Quincke needle, the long Whitacre spinal needle extends approximately 1.1 cm beyond the Quincke spinal needle (Figure 1). This relationship is specific to this particular set of BD spinal needles, so if different needles are used, it is essential for the anesthesia provider to verify the compatibility for needle-through-needle entry and measure the extent of needle advancement relative to the other.

Assuming the midline trajectory toward the interlaminar space is correct, the absence of CSF in the Whitacre spinal needle at a certain depth indicates that the needle has not yet reached the dural sac. In this situation, the Whitacre needle should be withdrawn (or pulled back inside the Quincke “Super Introducer”), allowing for the safe advancement of the 3.5” Quincke “Super Introducer” by approximately 0.8 cm without risking dural sac entry.

The choice of 0.8 cm rather than 1 cm provides a safety margin to prevent accidental insertion of the larger bore 20G Quincke needle into the thecal sac. If the provider finds it challenging or concerning to precisely measure 0.8 cm, advancing the needle by smaller increments, such as 0.5 cm, can enhance comfort and caution. However, this approach may require more steps to reach the intrathecal space.

To further control the advancement of the Quincke needle, the provider can pinch the needle approximately 0.8 cm (or less) from the skin entry point of the 20G Quincke needle during each advancement. The advancement should be stopped when the pinched fingers come into contact with the skin on the patient's back.

**Figure 2:**
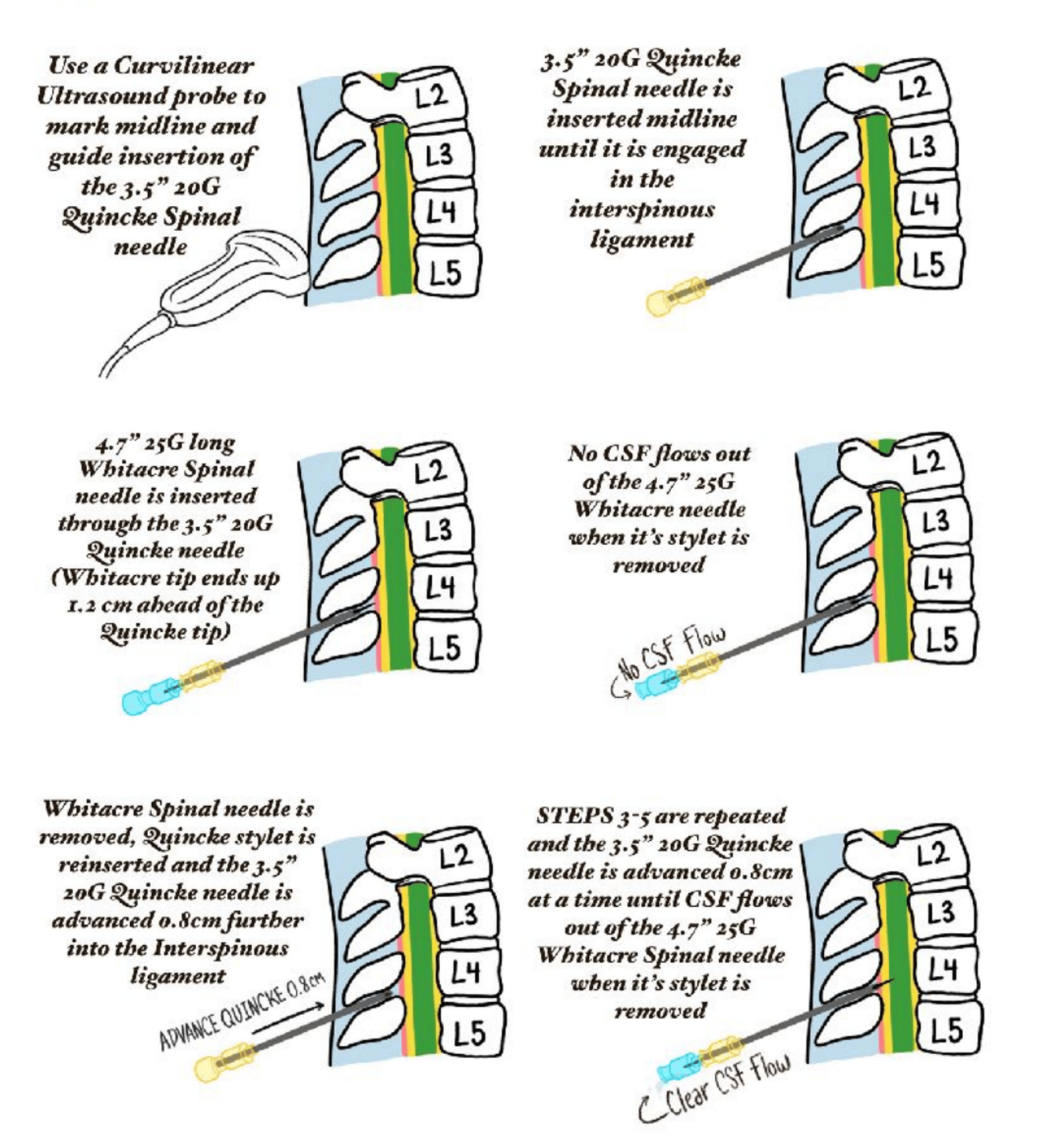
Animated illustration of the methodical step-by-step approach for safely performing the NNG technique using a “Super Introducer” NNG, needle-over-needle guidance Illustration developed by Phoenix Rose Hoffman based on draft drawings by Mahesh Desilva

After advancing the 3.5” 20G Quincke “Super Introducer,” the 4.7” Whitacre needle can be reinserted through the Quincke to approach the dural sac. This process is repeated until CSF is obtained in the Whitacre needle (Figure 2). If bone is encountered, the spinal attempt should be repeated with a different trajectory using the same methodical, step-by-step NNG approach to avoid os and successfully enter the dural sac.

A potential complication of the “Super Introducer” NNG technique is the inadvertent entry of the 20G Quincke needle into the dural sac, which significantly increases the risk of PDPH, particularly in younger patients. To minimize this risk, the anesthesia provider must exercise caution and accurately advance the Quincke “Super Introducer” by a maximum of approximately 0.8 cm at a time.

Using a 22G Quincke needle as the “Super Introducer” (paired with a long 27G-29G spinal needle) could further reduce the risk of PDPH if accidental dural sac entry occurs, compared to a 20G Quincke needle. Additionally, commercially produced 20G-22G Quincke spinal needles with 1 cm markings (similar to Tuohy epidural needles) would improve the accuracy of advancing the “Super Introducer” during this technique.

Another drawback of the “Super Introducer” NNG technique is the multiple advancements and withdrawals of the needles and needle stylets, which adds time to the procedure and increases the risk of accidental dropping or contamination. Therefore, careful attention to sterile technique and proper needle handling is essential. It is also crucial not to redirect the Quincke “Super Introducer” while the longer Whitacre spinal needle is advanced beyond it, or if bone is encountered at the tip of the Whitacre needle, to reduce the risk of needle fracture or breakage.

**Figure 3:**
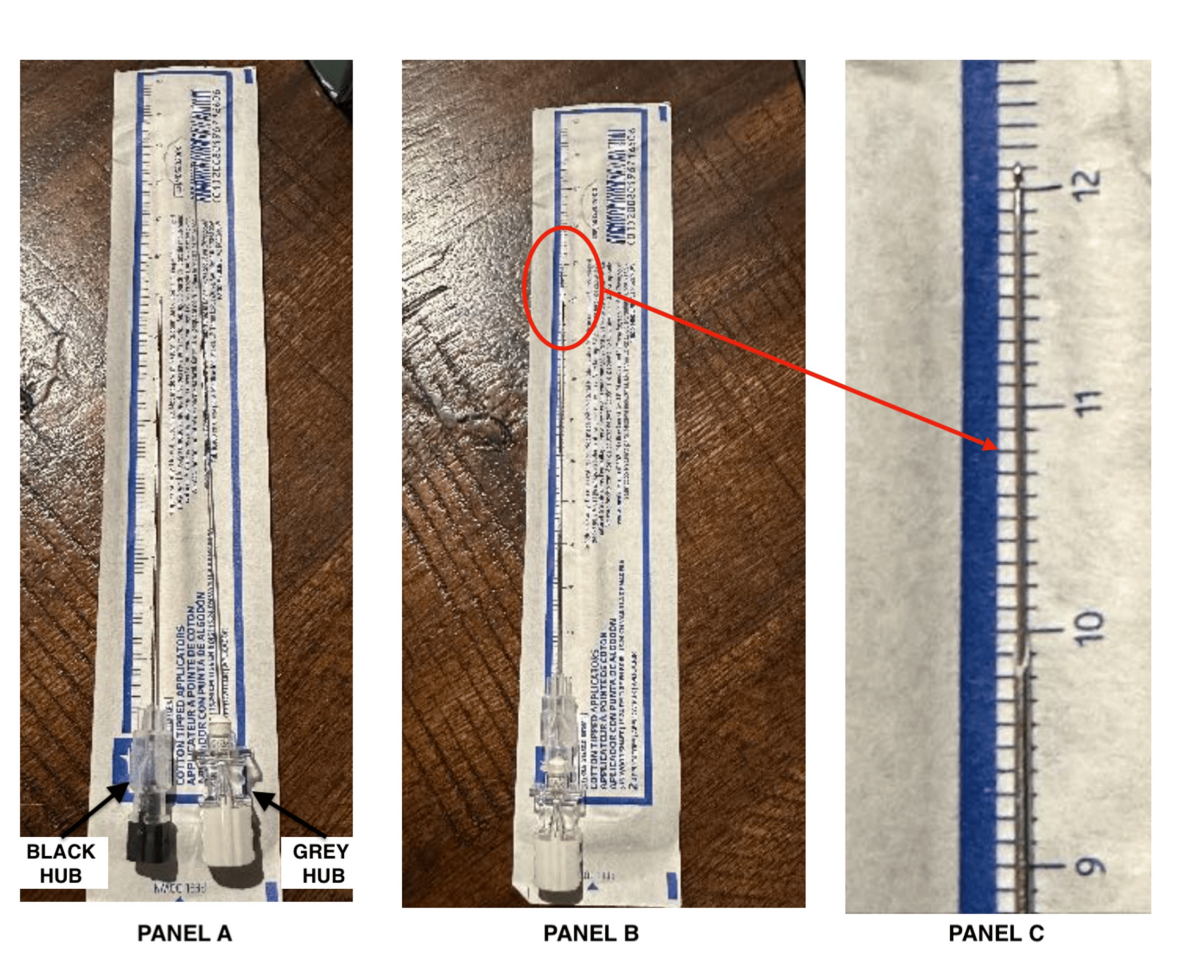
Alternative needle combination for the NNG technique using a “Super Introducer” A 5” (12.7 cm) 27G B Braun PENCAN spinal needle (grey hub) is used over a regular 3.5” (9 cm) 22G BD Quincke spinal needle (black hub) introducer. When inserted fully hub to hub, the long PENCAN spinal needle extends approximately 2 cm beyond the Quincke spinal needle. (A) 3.5” (9 cm) 22G BD Quincke spinal needle (black hub) and 5” (12.7 cm) 27G B Braun PENCAN spinal needle (grey hub) laid side by side. (B) 5” (12.7 cm) 27G B Braun PENCAN spinal needle fully inserted through the 3.5” (9 cm) 22G BD Quincke "Super Introducer" spinal needle. (C) When inserted completely hub to hub, the 5” B Braun PENCAN spinal needle extends approximately 2 cm beyond the 3.5” BD Quincke spinal needle. NNG, needle-over-needle guidance

An alternative combination for the “Super Introducer” NNG technique is to use a long 5” (12.7 cm) 27G PENCAN B Braun needle or a 5” 29G SPINOCAN B Braun needle over a regular 3.5” (9 cm) BD 22G Quincke needle introducer. When fully inserted through the 3.5” Quincke, the long 5” 27G PENCAN spinal needle extends approximately 2 cm beyond the Quincke spinal needle (Figure 3). This combination may be preferable for younger patients and obstetric cases, as accidental dural puncture with a 22G Quincke needle is less likely to cause PDPH than with a 20G Quincke, as used in this case report.

**Figure 4:**
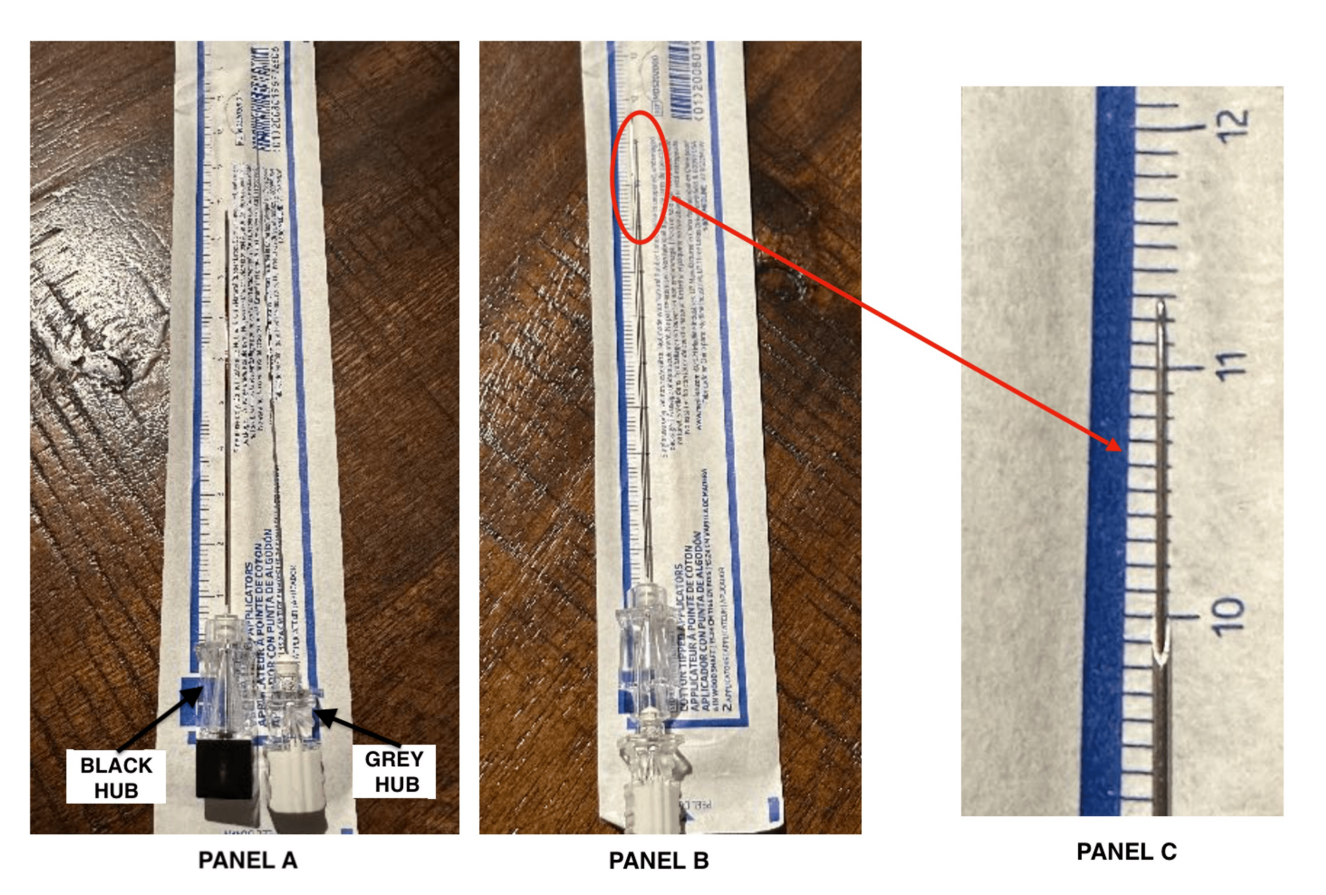
Alternate needle combination for the NNG technique using a “Super Introducer” with a 5” (12.7 cm) 27G B Braun PENCAN spinal needle (grey hub) over a regular 3.5” (9 cm) 22G Reli Quincke spinal needle (black hub) introducer Due to the larger hub of the 22G Reli Quincke compared to the 22G BD Quincke needle used in Figure 3, the PENCAN spinal needle advances only approximately 1.2 cm beyond the Reli Quincke “Super Introducer” when inserted hub to hub. This highlights the importance of understanding the needle combination used for the NNG technique. (A) 3.5” (9 cm) 22G Reli Quincke spinal needle (black hub) and 5” (12.7 cm) 27G B Braun PENCAN spinal needle (grey hub) laid side by side. (B) 5” (12.7 cm) 27G B Braun PENCAN spinal needle fully inserted through the 3.5” (9 cm) 22G Reli Quincke “Super Introducer” spinal needle. (C) When fully inserted hub to hub, the 5” B Braun PENCAN spinal needle extends approximately 1.2 cm beyond the 3.5” Reli Quincke spinal needle. NNG, needle-over-needle guidance

An important aspect of the “Super Introducer” NNG technique is understanding the relationship between the paired needles, which determines how far the Quincke “Super Introducer” needle can be advanced with each step. For example, when using a Reli 22G 3.5” (9 cm) Quincke needle with its larger hub alongside a long 27G 5” (12.7 cm) PENCAN needle, the PENCAN needle extends approximately 1.2 cm beyond the Quincke needle (Figure 4). This is in contrast to the approximately 2 cm advancement achieved when using a 22G 3.5” BD Quincke needle (Figure 3).

## Conclusions

The NNG technique using a “Super Introducer” offers a safe and effective approach for anesthesia providers to enhance spinal anesthetic placement. This technique is particularly valuable when increased depth to the dural sac and a higher risk of PDPH are expected, such as in morbidly obese younger patients who would benefit from the use of a longer pencil-point spinal needle to access the dura.
